# Expression of Tissue factor in Adenocarcinoma and Squamous Cell Carcinoma of the Uterine Cervix: Implications for immunotherapy with hI-con1, a factor VII-IgGF_c _chimeric protein targeting tissue factor

**DOI:** 10.1186/1471-2407-11-263

**Published:** 2011-06-22

**Authors:** Emiliano Cocco, Joyce Varughese, Natalia Buza, Stefania Bellone, Michelle Glasgow, Marta Bellone, Paola Todeschini, Luisa Carrara, Dan-Arin Silasi, Masoud Azodi, Peter E Schwartz, Thomas J Rutherford, Sergio Pecorelli, Charles J Lockwood, Alessandro D Santin

**Affiliations:** 1 Department of Obstetrics, Gynecology & Reproductive Sciences, Yale University School of Medicine, 333 Cedar street, New Haven (06519), CT, USA; 2 Department of Pathology, Yale University School of Medicine, 333 Cedar street, New Haven (06519), CT, USA; 3 Division of Gynecologic Oncology, University of Brescia, 1 Spedali Civili (52100), Brescia, Italy

## Abstract

**Background:**

Cervical cancer continues to be an important worldwide health problem for women. Up to 35% of patients who are diagnosed with and appropriately treated for cervical cancer will recur and treatment results are poor for recurrent disease. Given these sobering statistics, development of novel therapies for cervical cancer remains a high priority. We evaluated the expression of Tissue Factor (TF) in cervical cancer and the potential of hI-con1, an antibody-like-molecule targeted against TF, as a novel form of immunotherapy against multiple primary cervical carcinoma cell lines with squamous- and adenocarcinoma histology.

**Methods:**

Because TF is a transmembrane receptor for coagulation factor VII/VIIa (fVII), in this study we evaluated the *in vitro *expression of TF in cervical carcinoma cell lines by immunohistochemistry (IHC), real time-PCR (qRT-PCR) and flow cytometry. Sensitivity to hI-con1-dependent cell-mediated-cytotoxicity (IDCC) was evaluated in 5-hrs-^51^chromium-release-assays against cervical cancer cell lines *in vitro*.

**Results:**

Cytoplasmic and/or membrane TF expression was observed in 8 out of 8 (100%) of the tumor tissues tested by IHC and in 100% (11 out of 11) of the cervical carcinoma cell lines tested by real-time-PCR and flow cytometry but not in normal cervical keratinocytes (*p *= 0.0023 qRT-PCR; *p *= 0.0042 flow cytometry). All primary cervical cancer cell lines tested overexpressing TF, regardless of their histology, were highly sensitive to IDCC (mean killing ± SD, 56.2% ± 15.9%, range, 32.4%-76.9%, *p *< 0.001), while negligible cytotoxicity was seen in the absence of hI-con1 or in the presence of rituximab-control-antibody. Low doses of interleukin-2 further increased the cytotoxic effect induced by hI-con1 (*p *= 0.025) while human serum did not significantly decrease IDCC against cervical cancer cell lines (*p *= 0.597).

**Conclusions:**

TF is highly expressed in squamous and adenocarcinoma of the uterine cervix. hI-con1 induces strong cytotoxicity against primary cervical cancer cell lines overexpressing TF and may represent a novel therapeutic agent for the treatment of cervical cancer refractory to standard treatment modalities.

## Background

Despite aggressive screening programs, cervical cancer remains an important public health issue. In the United States about 12,200 new cases of cervical cancer as well as 4,210 deaths from cervical cancer are estimated for 2010 [1]. Although cervical cancer is, to a large extent, a preventable disease, it remains an important health problem for women, especially in underserved and minority groups in industrially developed nations and women in developing countries without established screening programs. While early stage cervical cancer can be cured by radical surgery or radiotherapy with equal effectiveness [2], pelvic radiation represents the standard therapy for the treatment of locally advanced disease. Despite technological advances, however, up to 35% of patients overall will develop recurrent disease, for which treatment results are poor [3]. A deeper understanding of the molecular basis of cervical cancer has the potential to significantly refine the diagnosis and management of these tumors and may eventually lead to the development of novel, more specific, and more effective treatments for prevention of disease progression following first-line therapy.

Angiogenesis, the formation of new vessels from pre-existing vasculature, is known to represent a critical step in the development, progression and metastatic process of human solid tumors. Tissue factor (TF), a transmembrane receptor for coagulation factor VII/VIIa (fVII), is aberrantly expressed in human cancers and on endothelial cells within the tumor vasculature [4,5]. Importantly, tumor cells characterized by a high production of TF and vascular endothelial growth factor (VEGF), a crucial initiator of angiogenesis, are known to generate solid tumors characterized by intense vascularity and highly aggressive behavior [6]. Consistent with this view, several studies have shown that VEGF is overexpressed and secreted in a variety of human tumors including cervical carcinomas [7] and an elevated expression of VEGF is correlated clinically with cervical cancer metastasis and poor patient survival [8]. While a direct regulation of VEGF expression in human tumor cells by the cytoplasmic tail of TF has been previously demonstrated [7], recent studies indicate that type-2 proteinase activated receptor (PAR-2) is intimately involved in TF-mediated signaling and angiogenesis [9]. These data suggest a potential direct role for TF in tumor growth [9].

hI-con1™ (Iconic Therapeutics, Inc.; Atlanta, GA) is a previously characterized immuno-conjugate molecule developed against TF [10-12]. It is composed of two identical protein chains consisting of human fVII as the targeting domain fused to human IgG_1 _F_c _as the effector domain; the two chains are held together by the disulfide bonds normally present in IgG. The hI-con1 is designed to bind to TF with far higher affinity and specificity than can be achieved with an anti-TF antibody. Indeed, the hI-con1 has several important advantages over monoclonal antibodies for targeting TF including: 1) The K_d _for fVII domain binding to TF is about 10^-12 ^M [13], in contrast to anti-TF antibodies that have a K_d _in the range of 10^-8 ^to 10^-9 ^M for TF [14], and 2) the hI-con1 is produced by recombinant DNA technology, allowing a completely human hI-con1 to be made for future clinical trials. Because binding of fVII to TF could induce disseminated intravascular coagulation, a potentially lethal vascular disease, an amino acid substitution was introduced into the fVII domain of the hI-con1 (Lys 341 to Ala) to inhibit initiation of the coagulation pathway without reducing the strong affinity for TF [10,15]. The human F_c _domain of the hI-con1 may thus potentially activate powerful cytolytic responses mediated by antibody-dependent cell-mediated cytotoxicity (ADCC) against both TF-expressing tumor cells and tumor vascular endothelial cells that bind the hI-con1 molecule.

To our knowledge, no studies have specifically described the expression of tissue factor in cervical cancer. Therefore, in this report we investigated the expression of TF at mRNA and protein levels in multiple primary cervical tumor cell lines and evaluated for the first time the *in vitro *potential of hI-con1 as a novel immunotherapeutic agent against biologically aggressive cervical cancer cell lines overexpressing TF.

## Methods

### Establishment of Primary Cervical Cancer Cell Lines

Primary cervical tumor cell lines from eleven patients harboring cervical cancer were obtained from fresh tumor biopsies collected at the time of primary surgery or surgery for cancer recurrence under approval of the Institutional Review Board. Tumors were staged according to the International Federation of Gynecologists and Obstetricians staging system. Source-patient characteristics of these eleven cell lines are described in Table 1. Human papillomavirus (HPV) typing of these specimens was performed by PCR using sequence-specific primers for HPV 16 and 18 against E7 oncoprotein (Integrated DNA Technologies, Coralville, IA), as previously described [16].

**Table 1:** Patient characteristics from which the 11 primary cancer cell lines were established

***Cell line***	***Age (years)***	***Race***	***FIGO Stage***	***Grade***	***Histopathology***
**ADX-1 ARK-1**	25	C*	IB	G3	Adenocarcinoma

**ADX-2 ARK-2**	33	C	IB	G3	Adenosquamous
**ADX-3 ARK-3**	33	AA**	IB	G3	Adenosquamous

**ADX-4 ARK-4**	46	C	IB	G2/G3	Adenocarcinoma
**CVX-1 ARK-1**	35	AA	IB	G3	Squamous

**CVX-2 ARK-2**	40	C	Recurrence	G3	Squamous
**CVX-3 ARK-3**	70	C	IB	G3	Squamous

**CVX-4 ARK-4**	40	C	IB	G3	Squamous
**CVX-5 ARK-5**	22	C	IB	G3	Squamous

**CVX-6 ARK-6**	29	C	IB	G3	Squamous
**CVX-7 ARK-7**	42	C	Recurrence	G3	Squamous

^FIGO, International Federation of Gynecology and Obstetrics

*C, Caucasian;

**AA, African-American

### Quantitative real-time PCR in Primary Cell Lines

RNA isolation from 11 primary cervical cancer cell lines (Table 1) was performed using TRIzol Reagent (Invitrogen, Carlsbad, CA) according to the manufacturer's instructions. Quantitative PCR was done with a 7500 Real Time PCR System using the manufacturer's recommended protocol (Applied Biosystems, Foster City, CA) to evaluate expression of TF in all samples. Each reaction was run in duplicate. Briefly, 5 μg of total RNA from each sample was reverse transcribed using SuperScript III first-strand cDNA synthesis (Invitrogen). Five μL of reverse transcribed RNA samples (from 500 μL of total volume) were amplified by using the TaqMan Universal PCR Master Mix (Applied Biosystems) to produce PCR products specific for TF. The primers and probe for TF were obtained from Applied Biosystems (Assay ID Hs01076032_m1). The comparative threshold cycle (*C*_T_) method (Applied Biosystems) was used to determine gene expression in each sample relative to the value observed in the lowest nonmalignant cervical keratinocyte cell sample, using glyceraldehyde-3-phosphate dehydrogenase (Assay ID Hs99999905_m1) RNA as internal control.

### TF immunostaining of Formalin-fixed Cervical Cancer Tissues

Formalin-fixed, paraffin-embedded tissue blocks obtained from 5 stage IB patients, 1 stage IIA and 2 stage III B patients (i.e., 4 squamous- and 4 adenocarcinomas) were retrieved from a separate set of 8 patients from the surgical pathology files at Yale University. All tumor samples and eight normal cervical tissue specimens used as controls were reviewed by a surgical pathologist (NB). The level of TF expression was then evaluated on the most representative block by standard immunohistochemical (IHC) staining. For immunohistochemistry, 4 μm sections were cut from the formalin-fixed paraffin-embedded blocks. Following deparaffinization and rehydration, endogenous peroxidase was blocked in 3% H_2_O_2_. Steam and high pH (pH 9) were used for antigen retrieval. The slides were then incubated overnight at 4°C with monoclonal anti-TF antibody (No.4509, 1:10 dilution, American Diagnostica, Stamford, CT). EnVisionTM system (Dako, Carpinteria, CA) was used for secondary detection and the reactions were visualized with diaminobenzidine. Appropriate positive and negative controls were used with each case. Both cytoplasmic and membranous immunoreactivity was considered positive. Immunostaining was assessed using a semi-quantitative scoring system, as follows: 0, negative (0% to 5% staining); 1+, weakly positive (10% to 20%); 2+, moderately positive (20% to 50%); 3+ and 4+, strongly positive (50% to 75% and over 75%, respectively), as previously described [17].

### Flow Cytometry

Clinical grade hI-con1 was produced for Iconic Therapeutics Inc. (Atlanta, GA) by Laureate Pharma, (Princeton, NJ) by cultivation of BHK cells transfected with a vector containing the gene sequence originally described by Hu and Garen [10-12]. The protein was purified by a series of chromatographic steps to a purity adequate for clinical use, formulated in 15 mM HEPES, 150 mM NaCl, 5 mM CaCl_2_, 25 mM arginine, 0.01% Tween 80, pH 7.4 buffer and sterile-filtered. It was provided in aliquots of 80 μL at a stock concentration of 350 μg/ml which were used for our flow cytometry and hI-con1-dependent cell-mediated cytotoxicity (IDCC) studies. Briefly, cervical cancer cell lines and phytohemagglutinin (PHA)-stimulated control peripheral blood lymphocytes (PBL) were stained with hI-con1 at a concentration of 30 μg/ml for 30 minutes on ice or with a commercially available FITC-conjugated mouse anti-human TF immunoglobulin (i.e., positive control, BioSource International, Camarillo, CA). After hI-con1 staining, cells were washed twice with the same buffer and secondary mouse-anti-human antibody (IgG_1_-FITC, catalog # F0767 Sigma Aldrich, S. Louis, MO) was added for a further 30 minutes. Analysis was conducted with a FACSCalibur instrument using Cell Quest software (Becton Dickinson, Franklin Lakes, NJ).

### Tests for IDCC

A standard 5-hours chromium (^51^Cr) release assay was performed to measure the cytotoxic reactivity of Ficoll-Hypaque-separated PBL from several healthy donors in combination with hI-con1 against cervical cancer cell lines. The release of ^51^Cr from the target cells was measured as previously described [17] as evidence of tumor cell lysis after exposure of tumor cells to various concentrations of hI-con1 (ranging from 1 to 80 μg/ml) and different target/effector cells ratios. Controls included the incubation of target cells alone or with PBL or monoclonal antibody (mAb) separately. The chimeric anti-CD20 mAb rituximab (Rituxan^®^; Genentech) was used as control in all bioassays. IDCC was calculated as the percentage of killing of target cells observed with mAb plus effector cells as compared with ^51^Cr release from target cells incubated alone.

### IL-2 Enhancement of IDCC

To investigate the effect of interleukin-2 (IL-2) on IDCC, effector PBL were incubated for 5 hours at 37°C at a final concentration of IL-2 (Aldesleukin; Chiron Therapeutics, Emeryville, CA) ranging from 50 to 100 IU/ml in 96-well microtiter plates. Target cells were primary cervical cancer cell lines exposed to hI-con1 (concentrations ranging from 1 to 80 μg/ml), whereas controls included the incubation of target cells alone or with PBL in the presence or absence of IL-2 or mAb, respectively. Rituximab was used as a control mAb. IDCC was calculated as the percentage of killing of target cells observed with mAb plus effector PBL, as compared with target cells incubated alone. Each experiment was performed with PBL collected from multiple normal donors, with results from a representative donor presented.

### Test for complement-mediated target cell lysis and γ-globulin inhibition

A standard five-hour chromium (^51^Cr) release assay identical to the previous IDCC assays was used, except that human serum in a dilution of 1:2 was added to the effector cells. This human serum was used as a source of complement to test for complement-mediated target cell lysis. To test for the possible inhibition of IDCC against a chemoresistant squamous cervical cancer cell line by physiological human serum concentrations of γ-globulin, human serum was diluted 1:2 before being added in the presence or absence of effector PBL. Experiments were also performed using heat-inactivated human serum (56°C for 60 minutes; diluted 1:2) in the presence of effector PBL. Controls included the incubation of target cells alone or with either PBL or mAb separately. Rituxan was used as a control mAb.

### Statistical Analysis

For qRT-PCR data, the right-skewing was removed by taking copy-number ratios relative to the lowest-expressing normal cervical keratinocyte sample ("relative copy numbers"), log_2_-transforming them to ΔCTs, and comparing the results via unequal-variance *t*-test for differences between cervical cancer cells versus normal cervical keratinocytes. Group means with 95% confidence intervals (CI) were calculated by computing them on the ΔCTs and then reverse-transforming the results to obtain means (95% CI) of relative copy numbers. Differences in TF expression by IHC and flow cytometry were analyzed by the unpaired t-test. Kruskal-Wallis test and chi-square analysis were used to evaluate differences in hI-con1-dependent cell-mediated cytotoxicity levels in primary tumor cell lines. Statistical analysis was performed using SPSS version 17 (SPSS, Chicago, IL). A *p*-value of < 0.05 was considered statistically significant.

## Results

### Tissue Factor expression by qRT-PCR in primary cell lines

A total of eleven primary cervical cancer cell lines including 7 squamous cervical tumors, 2 cervical adenocarcinomas and 2 adenosquamous tumors were available for this study (Table 1). All primary cervical cancer cell lines were found to harbor HPV 16 or 18 genotypes (data not shown). With the exception of CVX-1 ARK-1 and CVX-7 ARK-7 cervical cancer cell lines, which were derived from biopsies obtained from recurrent carcinoma sites (i.e., a vaginal recurrence, CVX-1 ARK-1) and a metastatic site of disease (CVX-7 ARK-7, i.e., lymph node), all other cell lines were established from biopsies collected at the time of surgery from the primary site of disease (i.e., cervix uteri, Table 1). The mean ± standard error (minimum-maximum) copy number in tumor samples was 224.8 ± 39.4 (range: 44-613) versus 0.5 (range: 0.02-1) in normal cells (*p *= 0.023). Of the eleven tumors tested, nine showed a high mRNA copy number (i.e., above 100 copies) (Table 2). Squamous tumor cell lines were found to express significantly higher level of TF (mean ± SE = 297.2 ± 51) when compared to the adenocarcinomas and adenosquamous tumor cell lines (mean ± SE = 134.2 ± 37.9) (*p *= 0.037, Table 2). Of interest, both tumor cell lines (i.e., CVX-1 ARK-1 and CVX-7 ARK-7) established from recurrent/metastatic cervical tumors were found to express high levels of TF mRNA copy numbers (i.e., 382 and 480, respectively, Table 2).

**Table 2:** Tissue Factor expression by qRT-PCR in primary squamous and adenocarcinoma cervical cancer cell lines

***Cell line***	***qRT-PCR***
	
	mRNA copy #
ADX-1 ARK-1	46.7
ADX-2 ARK-2	156.5
ADX-3 ARK-3	44.2
ADX-4 ARK-4	289.7
CVX-1 ARK-1	382.1
CVX-2 ARK-2	138.3
CVX-3 ARK-3	137.2
CVX-4 ARK-4	109.6
CVX-5 ARK-5	613.9
CVX-6 ARK-6	123.9
CVX-7 ARK-7	480.6

*qRT-PCR, quantitative Real-time Polymerase Chain Reaction.

### Tissue Factor expression by IHC in Cervical Carcinoma samples

**Figure 1:**
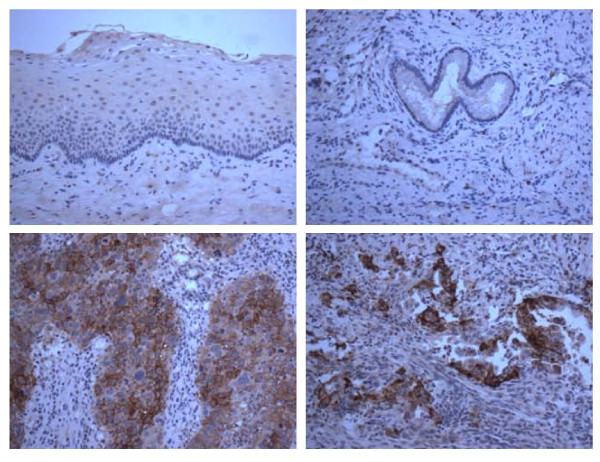
**Representative IHC of TF in squamous- and adenocarcinoma specimens versus normal cervical control tissues**. Upper left panel: normal ectocervical squamous epithelium negative for TF. Lower left panel: cervical squamous cell carcinoma showing high expression of TF. Upper right panel: normal cervical glands negative for TF. Lower right panel: cervical adenocarcinoma showing high expression of TF. Original magnification: 200×.

We performed immunohistochemical analysis of TF protein expression on formalin-fixed tumor tissues from 8 paraffin-embedded cervical cancer specimens including 4 squamous- and 4 adenocarcinomas. As representatively shown in Figure 1, we found TF expression in 4 out of 4 (100%) of the squamous carcinomas (i.e., one 4+, two 2+, and one 1+) and 4 out of 4 (100%) of the adenocarcinomas (i.e., one 3+, one 2+, and two 1+). The cervical cancer samples showed either membrane and/or cytoplasmic immunoreactivity for TF while the non-neoplastic cervical squamous epithelium and normal cervical glands were found consistently negative for TF expression (Figure 1).

### Tissue Factor expression by flow cytometry in primary cervical carcinoma cell lines

**Figure 2:**
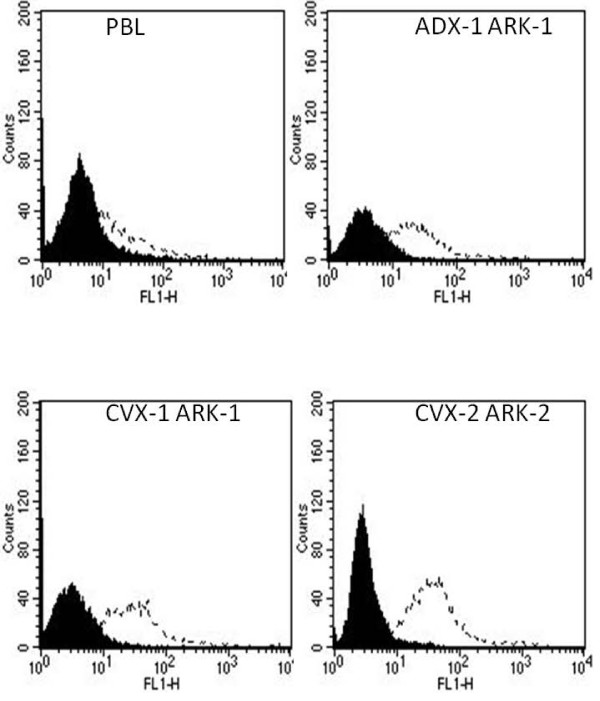
**Representative TF expression by flow cytometry of control PBL (negative control) and CVX-1 ARK-1, CVX-2 ARK-2 and ADX-1 ARK-1 primary cervical cell lines**. Isotype (solid black); hI-con1 (dashed line).

Surface TF receptor expression was evaluated by FACS analysis on all eleven primary cervical cancer cell lines using hI-con1 and an anti-human TF control mAb. As negative controls, several PHA-stimulated PBL established from healthy donors or the same cervical cancer patients from which the tumor cell lines had been established were also studied. Reactivity against TF was found by flow cytometry in all cervical cancer cell lines tested, with ADX-1 ARK-1 and ADX-3 ARK-3 showing lower expression levels when compared to all the remaining cells lines (Figure 2 and data not shown). Mean fluorescence intensity (MFI) for TF ranged from 61.3 to 106.7 (mean ± SE = 83.8 ± 7.7) in cervical tumor cell lines versus a MFI ± SE of 35.01 ± 7.5 in the PHA-stimulated PBL populations used as negative controls (Figure 2, *p *= 0.0042).

### Cervical tumors overexpressing TF are sensitive to hI-con1-dependent cell mediated cytotoxicity (IDCC)

**Figure 3:**
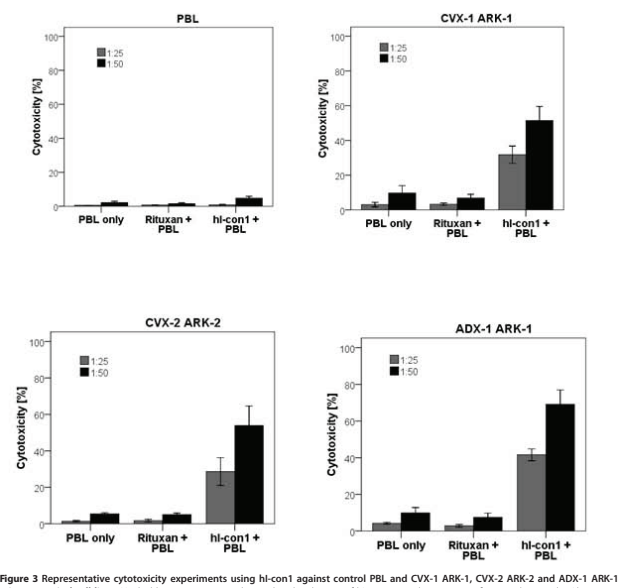
**Representative cytotoxicity experiments using hI-con1 against control PBL and CVX-1 ARK-1, CVX-2 ARK-2 and ADX-1 ARK-1 primary cervical cell lines**. Negligible cytotoxicity was detected in the absence of hI-con1 or in the presence of rituximab control mAb.

Four cervical cancer cell lines, ADX-1 ARK-1 and CVX-2 ARK-2 (established from biopsies obtained from a primary adenocarcinoma and a squamous tumor, respectively), and CVX-1 ARK-1 and CVX-7 ARK-7 (established from recurrent squamous cervical carcinomas), were tested for their sensitivity to natural killer cell (NK) cytotoxicity when challenged with heterologous PBL, collected from several healthy donors, in standard 5-hours ^51^Cr release assay. All primary cervical cancer cell lines were consistently found to be resistant to NK-mediated cytotoxicity when combined with PBL at effector to target (E:T) ratios of 25:1 and 50:1 (range of cytotoxicity: 0.5 - 17.6% with all E:T ratios). Similarly, primary cervical cancer cell lines incubated with rituximab (2.5 μg/ml) control antibody displayed no significant cytotoxicity (range: 0.5% - 13.03%). In contrast, as representatively shown in Figure 3, we found all cervical tumor cell lines tested expressing TF, regardless of their squamous or adenocarcinoma histology or their primary versus metastatic/recurrent tumor sites of origin, to be highly sensitive to IDCC (mean ± SD: 56.2% ± 15.9%, range: 32.4% - 76.9% for the E:T ratio 50:1, Figure 3, *p *< 0.001). Importantly, high levels of hI-con1-induced cytotoxicity were also detected against CVX-1 ARK-1, a squamous cervical cancer cell line established from a patient experiencing recurrent disease after chemo-radiation resistant tumor (Figure 3) and CVX-7 ARK-7, a tumor established from a metastatic recurrent site of disease (data not shown). Negligible cytotoxicity was detected against PHA-stimulated PBL controls incubated with hI-con1 (mean ± SD, 4.8% ± 1.8% for the E:T ratio 50:1; Figure 3).

### IL-2 Enhancement of IDCC against cervical tumors

**Figure 4:**
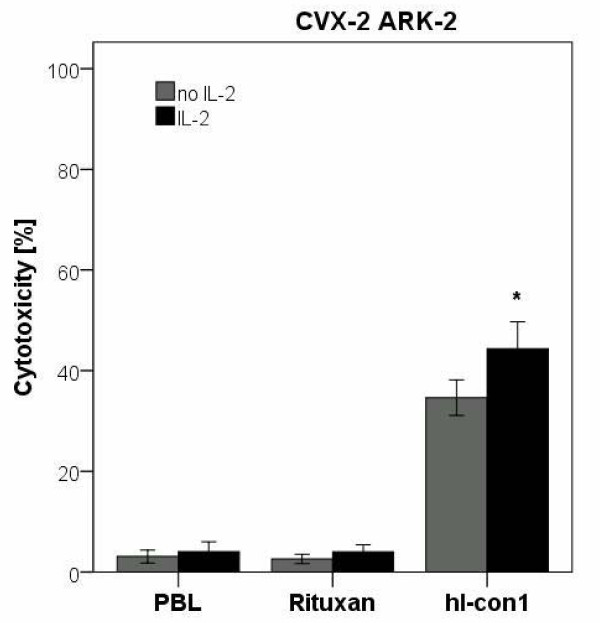
**Representative effect of low doses of interleukin-2 (IL-2) in combination with hI-con1 (30 μg/ml) on ADCC against CVX-2 ARK-2 primary cell line (Effectors to target ratio 50:1 and 25: 1)**. PBL from healthy donors were incubated for 5 hours in the presence of 100 IU/ml of IL-2. hI-con1-DCC was significantly increased in the presence of low doses of IL-2. No significant increase in cytotoxicity was detected after 5-h IL-2 treatment in the absence of hI-con1 or in the presence of the rituximab isotype control mAb.

To investigate the effect of low doses of IL-2 in combination with hI-con1 (30 μg/ml) on IDCC against primary cervical cancer cell lines, PBL from healthy donors were incubated for 5 hours in the presence of 50 to 100 IU/ml of IL-2. As representatively shown in Figure 4 for CVX-2 ARK-2, in multiple experiments in all cervical tumors tested in the presence of IL-2 (i.e., ADX-1 ARK-1, CVX-1 ARK-1 and CVX-2 ARK-2), IDCC was consistently increased in the presence of low doses of the cytokine. Administration of 50 to 100 IU/ml of IL-2 to the effector PBL at the start of the assay increased the cytotoxic activity against primary cervical cancer cell lines overexpressing TF compared with the use of hI-con1 alone (Figure 4, *p *= 0.025) whereas no significant increase in cytotoxicity was detected after 5 hours of IL-2 treatment in the absence of hI-con1 or in the presence of rituximab control mAb (Figure 4). These results demonstrate that low levels of IL-2 may enhance hI-con1-mediated-cell-cytotoxicity in primary cervical cancer cell lines *in vitro*.

### Effect of complement and physiological concentrations of IgG on hI-con1-mediated IDCC

**Figure 5:**
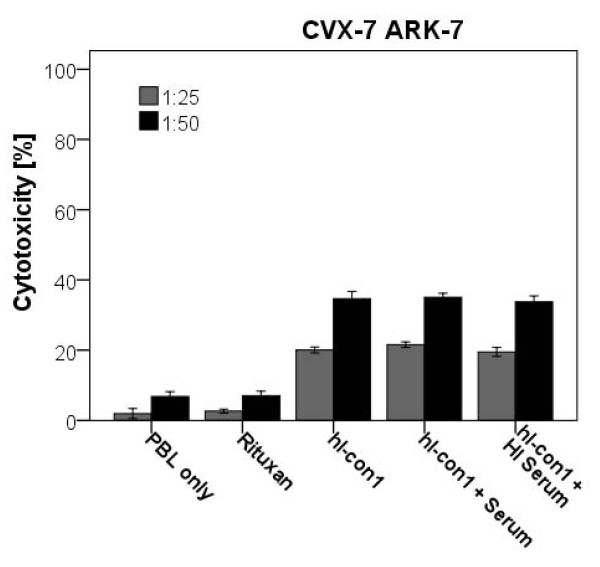
**Cytotoxicity experiments adding human serum to hI-con1 (30 μg/ml) against one representative primary cervical cancer cell line overexpressing TF (i.e., CVX-7 ARK-7: effectors to target ratio 50:1 and 25:1)**. Cervical cancer cells were challenged by diluted (1:2) serum (with or without heat inactivation) added in the presence of the effector cells and hI-con1 in standard 5-h ^51^Cr release assays. Addition of treated or untreated human serum (diluted 1:2) to PBL in the presence of hI-con1 did not significantly change hI-con1-mediated cytotoxicity against CVX-7 ARK-7 (*p *= 0.597).

In order to evaluate the effect of complement on the hI-con1-mediated IDCC as well as the potential inhibition of hI-con1 cytotoxic activity by physiological human serum γ-globulin concentrations, human serum diluted 1:2 (with and without heat inactivation) was added to a representative squamous cervical cancer cell line (CVX-7 ARK-7) during standard 5-hours ^51^Cr release assays. The addition of untreated or heat inactivated human serum diluted 1:2 did not significantly alter the cytotoxic effect of hI-con1 against CVX-7 ARK-7 cell line when compared to hI-con1-mediated killing without serum (*p *= 0.597, Figure 5).

## Discussion

The management of disseminated carcinoma of the cervix that is no longer amenable to control with surgery or radiation therapy has not improved significantly with the advent of modern chemotherapy [2,3]. The one-year survival rate remains between 10 and 15 percent [2,3]. Thus, the development of novel, target-specific and effective modalities against cervical cancer refractory to standard treatments remains desperately needed.

We have evaluated Tissue Factor (TF) expression by qRT-PCR, IHC and flow cytometry in cervical tissue samples and multiple primary cervical cancer cell lines with squamous- and adenocarcinoma histology, some of which were derived from recurrent chemo-radiation resistant tumors. In addition, we have tested the *in vitro *activity of hI-con1, a previously characterized immuno-conjugate molecule developed against TF [10-12], as a novel therapy against multiple primary cervical cancer cell lines *in vitro*. We found both squamous- and adenocarcinoma cell lines to overexpress TF when compared to normal cervical keratinocytes. We speculate that TF expression may be a common and important event in malignant transformation of the cervix into biologically aggressive squamous and adenocarcinoma cervical cancers. Furthermore, both cell lines available to this study established from recurrent/metastatic tumors were found to express extremely high levels of TF. These data are consistent with previous reports in other human solid tumors showing that a high production of TF and vascular endothelial growth factor (VEGF), generate solid tumors characterized by highly aggressive biologic behavior [6]. In this regard, TF is known to be involved in pathological angiogenesis and is abnormally overexpressed in multiple human tumors and in tumor vascular endothelial cells but not on normal quiescent vascular endothelial cells [5,6]. Although TF, as a cell surface receptor, is physiologically expressed on extravascular cells of many organs and in the adventitial layer of the blood vessel wall, it is sequestered by coagulation factor VII (fVII), a natural ligand for TF, at these sites by the tight endothelial cell layer of the normal vasculature [5,6]. Thus, pathologically expressed TF may provide a target for the development of novel cancer therapies effective not only against tumor cells but also tumor blood vessels [10-12,18].

To our knowledge, our results are the first to describe self-production of TF-coagulation factor VII complex by primary cervical cancer cell lines. These data are however consistent with multiple previous reports demonstrating an association between cervical cancer and deep-vein thrombosis and/or pulmonary thromboembolism (i.e., Trousseau syndrome) [19-22], a pathologic state previously reported to be associated with TF overexpression [23]. Importantly, primary cervical cancer cell lines overexpressing TF, regardless of their histology or chemo-radiation therapy resistance, were found to be highly sensitive to hI-con1-mediated cytotoxicity *in vitro*.

TF is known to play an important role in tumor metastatic process, possibly by inducing the coating of the tumor cell with fibrin that would trap the cells in the microvasculature, thereby aiding metastases [24-26]. Recently, however, a possible direct role for TF in tumor growth has also been suggested by studies showing a dramatically reduced tumor growth in mice where a selective reduction in TF was achieved using small interfering RNA [26]. Of great interest, in these studies, the reduction of TF expression did not affect growth of the tumor cells *in vitro*, suggesting that TF-mediated enhancement of tumor growth requires a factor present *in vivo *that is not present when cells are grown *in vitro *[26]. A potential candidate to explain these findings is therefore fVIIa, which would form a TF:fVIIa complex on the surface of tumor cells *in vivo *leading to activation of type-2 proteinase activated receptor (PAR2) dependent signaling [9]. These findings combined with our results suggest that TF overexpression may potentially provide an additional growth advantage to biologically aggressive cervical cancers *in vivo*.

The potential cytotoxic activity of hI-con1 against human melanoma and prostate tumor cells has previously been demonstrated by Hu et al. [10-12]. In a recent study we extended their results by evaluating the cytotoxic potential of hI-con1 against multiple high grade, biologically aggressive, Type II endometrial cancer cell lines [17]. We found all endometrial carcinomas that showed high TF expression, regardless of their high or low HER2/neu expression, were highly susceptible to IDCC in the presence of effector cells [17]. In the current study we expanded our research work with hI-con1 to multiple primary cervical carcinoma cell lines with squamous and adenocarcinoma histology. It is worth noting that although these cell lines were resistant to natural killer cytotoxic activity, IDCC resulted in killing of up to 50% of tumor cells in 5-hour ^51^Cr release assays. Taken together, these *in vitro *results strongly suggest that TF may provide a novel target for the treatment of resistant/residual cervical disease and the destruction of their tumor vasculature that should result in hI-con1-induced lysis of tumor cells as well as tumor endothelial cells *in vivo*. Consistent with this hypothesis and the central role of angiogenesis in cervical cancer development and progression, bevacizumab, a humanized antibody able to bind and inactivate VEGF, has been recently reported to shrink cervical tumors and delay progression without appreciable toxicity. On the basis of these encouraging results, bevacizumab is currently being studied in a Gynecologic Oncology Group (GOG) phase III trial [27].

For effective cytotoxicity, the effector cells must be able to interact with the immune-conjugate at the target site *in vivo*, even in the presence of high concentrations of human IgG. In this study we showed that hI-con1-dependent cytotoxicity against cervical cancer cell lines was not inhibited by high concentrations (up to 50%) of human serum. These data, therefore, suggest that in the presence of effector PBL, human serum may not significantly decrease IDCC against cervical cancer cell lines. Moreover, these results indicate that the binding of hI-con1 to the F_c _receptor on mononuclear effector cells is likely to occur *in vivo*.

The degree of ADCC *in vitro *and *in vivo *is known to be mainly dependent on the number and function of NK cells [28]. NK cells are considered to be the best suited lymphocytes for ADCC because they carry F_c_γRIII receptors, which are activator molecules, and not F_c_γRIIb receptors, which inhibit ADCC [29]. NK cells also carry receptors for interleukin-2 (IL-2), a cytokine which induces expansion of the NK cell population and enhances their function [29-31]. Therefore, low dose IL-2 administered in the outpatient setting has been previously used to enhance cancer patients' immune response to monoclonal antibodies with little toxicity [30,31]. These findings are particularly interesting because in previous studies we have found that a major barrier to the successful development of therapeutic immunization of advanced cervical cancer patients harboring disease refractory to radiation and chemotherapy was represented by a profound state of immunosuppression present in the majority of these patients [32]. This anergic state, reported by other investigators who have also evaluated the immunocompetence of cervical cancer patients [33], has been attributed to the compounded negative effects of tumor-induced immunomodulation with those resulting from radiation and chemotherapy [34,35]. Importantly, our *in vitro *experiments reveal a significant increase of hI-con1-induced cytotoxicity after the brief incubation of PBL and tumor cells with IL-2 compared to the cytotoxicity induced by hI-con1 in the absence of IL-2. IL-2 seems to therefore enhance the cytotoxic potential of the effector cells. The administration of low doses of IL-2 might therefore be a valid therapeutic option in order to increase IDCC in heavily pretreated cervical carcinoma patients.

## Conclusions

In conclusion, high expression of TF in a large number of cervical carcinomas makes hI-con1 an attractive agent for immunotherapy of recurrent/refractory cervical cancer. In this study, we have demonstrated significant hI-con1-mediated killing of multiple primary cervical cancer cell lines regardless of their squamous or adenocarcinoma histology. hI-con1 might therefore represent a novel treatment of this aggressive disease and, potentially, multiple other human tumors overexpressing TF.

## Abbreviations

TF: tissue factor; fVII: Factor VII; hI-con1: human immuno-conjugate molecule; IDCC: hI-con1-dependent cell-mediated cytotoxicity; FBS: fetal bovine serum; IHC: immunohistochemistry; mAb: monoclonal antibody; NK cells: natural killer cells; PBL: peripheral blood lymphocytes; qRT-PCR: quantitative real-time-polymerase chain reaction; CVX: cervical squamous cell carcinoma; ADX: cervical adenocarcinoma.

## Competing interests

The authors declare that they have no competing interests.

## Authors' contributions

EC, JV, SB, MG, MB, PT, and LC carried out the molecular in vitro studies including RT-PCR, flow cytometry and IDCC assays, as well as statistical analysis. NB carried out the IHC studies on the tissue samples. DS, MA, PES, TJR, SP, CJL, and AS participated in the design of the study and drafted the manuscript. AS conceived the study. All authors read and approved the final manuscript.

## Pre-publication history

The pre-publication history for this paper can be accessed here:

http://www.biomedcentral.com/1471-2407/11/263/prepub
